# Hepatic transcriptome analysis and identification of differentially expressed genes response to dietary oxidized fish oil in loach *Misgurnus anguillicaudatus*

**DOI:** 10.1371/journal.pone.0172386

**Published:** 2017-02-17

**Authors:** Yin Zhang, Yang Li, Xiao Liang, Xiaojuan Cao, Longfei Huang, Jie Yan, Yanxing Wei, Jian Gao

**Affiliations:** 1 College of Fisheries, Key Lab of Agricultural Animal Genetics, Breeding and Reproduction of Ministry of Education/Key Lab of Freshwater Animal Breeding, Ministry of Agriculture, Huazhong Agricultural University, Wuhan, China; 2 Freshwater Aquaculture Collaborative Innovation Center of Hubei Province, Hubei, People’s Republic of China; Ohio State University, UNITED STATES

## Abstract

RNA sequencing and short-read assembly were utilized to produce a transcriptome of livers from loaches (*Misgurnus anguillicaudatus*) fed with three different diets respectively containing fresh fish oil (FO group), medium oxidized fish oil (MO group) and high oxidized fish oil (HO group). A total of 60,663 unigenes were obtained in this study, with mean length 848.74 bp. 50,814, 49,584 and 49,814 unigenes were respectively obtained from FO, MO and HO groups. There were 2,343 differentially expressed genes between FO and MO, with 855 down- and 1,488 up-regulated genes in the MO group. 2,813 genes were differentially expressed between FO and HO, including 1,256 down- and 1,552 up-regulated genes in the HO group. 2,075 differentially expressed genes were found in the comparison of MO and HO, including 1,074 up- and 1,001 down-regulated genes in the MO group. Some differentially expressed genes, such as *fatty acid transport protein* (*fatp*), *fatty acid binding protein* (*fabp*), *apolipoprotein* (*apo*), *peroxisome proliferator activated receptor-gamma* (*ppar-γ*), *acetyl-CoA synthetase* (*acs*) and *arachidonate 5-lipoxygenase* (*alox5*), were involved in lipid metabolism, suggesting these genes in the loach were responsive to dietary oxidized fish oil. Results of transcriptome profilings here were validated using quantitative real time PCR in fourteen randomly selected unigenes. The present study provides insights into hepatic transcriptome profile of the loach, which is a valuable resource for studies of loach genomics. More importantly, this study identifies some important genes responsible for dietary oxidized fish oil, which will benefit researches of lipid metabolism in fish.

## Introduction

There is a deficiency in desaturation and elongation pathways necessary for biosynthesis of eicosapentaenoic acid (EPA) and docosahexaenoic acid (DHA) in fish [1]. Supplementation of an adequate amount of highly unsaturated fatty acids (HUFAs) in diet is of great importance in maintaining their optimal health and growth [2]. Fish oil has now been the core in human health studies due to its large content of EPA and DHA. Fish oil is also the main lipid source of commercial aquatic diet for freshwater fish species. Nonetheless, fish oil is easy to be oxidized, which may result in forming toxic lipid hydroperoxides through a free radical chain reaction induced by trace metals, light or heat [3]. The lipid hydroperoxides which are regarded as the primary oxidation products can easily decompose to fatty acid alkoxy radicals [4] or even break down to release a series of secondary oxidation products such as aldehydes, ketones, alcohols and carboxylic acids [5], which are the main sources of unpleasant flavour and odour [6]. Fatty acid alkoxy radicals can oxidize most cellular constituents such as HUFAs, DNA, proteins and lipids [7]. Therefore, both lipid peroxidation and oxidation products are detrimental to dietary nutritive value and fish health as a result of oxidative lesions and damages. Undesirable influences of consuming oxidized dietary oil on fish metabolism have been observed in a number of studies [6, 8–14]. Interest in the dietary oxidized lipid associated with oxidative stress has grown in recent years, and researchers have become increasingly focused on the oxidative stress in cultured fish species [15–17]. At present, most of the researches about oxidized lipid in fish are focused on their growth or histological structures, while few on their molecular or genome profiles.

Recently, genome-wide gene expression profiling has gained ground by the development and application of large scale sequencing techniques to obtain gene sequences and develop molecular markers, especially in less researched species [18–22]. Sofia *et al* [23] found that dietary lipids could potentially affect some metabolic pathways based on transcriptome analysis. Sofia *et al* [24] also found the potential specific-genotypes in response to dietary vegetable oil through intestinal transcriptome analysis. Simona *et al* [25] inferred tissue-specific molecular signatures of lipid metabolism in fed and fasted fish according to the transcriptome analysis. There is no report on lipid metabolism of loach based on transcriptome analysis.

Therefore, in this paper we reported a hepatic transcriptome of loach *Misgurnus anguillicaudatus*, a small freshwater teleost that inhabits the muddy bottom of creeks, ponds, wetlands and paddy fields [26] and distributes widespreadly in China, Japan and other Southeast Asian countries [27], fed with three different diets, namely fresh fish oil diet (FO for short), medium oxidized fish oil diet (MO for short) and high oxidized fish oil diet (HO for short). This study systematically and quantitatively explored the effects of oxidized fish oil on the genome-wide gene expressions, which would provide insights into mechanisms of lipid metabolism in the loach. Meanwhile, the transcriptome obtained here will also provide useful information for future functional genomic studies in the loach.

## Materials and methods

### Ethics

All experiments were conducted in strict accordance with the recommendations in the Guide for the Care and use of Laboratory Animals of Huazhong Agricultural University. This study was approved by the Institutional Animal Care and Use Ethics Committee of Huazhong Agricultural University. During the 12 weeks feeding trial, loaches’ health statuses were monitored thrice daily (8:00, 14:00 and 20:00 h), in accordance with the feeding time. Throughout the entire feeding trial, all the loaches were healthy, in other words, no weak and sick loaches or injured-loaches were found and they were all active. After the feeding trial, the method of euthanasia (i.e. decapitation) was used for loaches anesthetized with 100 mg/L tricaine methanesulfonate (MS-222). And then the loaches were placed on ice for tissues collections. All efforts were made to minimize loach suffering.

### Diet formulation and preparation

Three experimental diets, namely FO, MO and HO, were used in this study. Compositions of the semi-purified basal diets are presented in S1 Table. Skimmed fishmeal was used as the major protein source and fish oil without any antioxidants as the lipid source. The fresh fish oil was oxidized by heating at 60°C with vigorous aeration constantly. Oxidized degree of the fresh fish oil monitored by the determination of the peroxide value (POV) was measured every 12 h until the POVs of the fish oils reached the needs of our experiment, i.e. 90.48 meq/kg for making MO and 313.58 meq/kg for making HO. The oxidized fish oils were then collected and stored at -20°C until using. After the fresh and oxidized fish oils mixed into diets, the POVs of the three diets were measured and they were 4.8 meq/kg (FO), 46.3 meq/kg (MO) and 96.0 meq/kg (HO), respectively. POVs of fish oils and diets were determined by the IUPAC Method [28]. Thiobarbiturate reactive substances (TBARS) of FO, MO and HO diet were 40.11, 78.16 and 98.14 μmol methane dicarboxylic aldehyde/mg diet, respectively. At the time of feeding, the diets were broken into appropriate sizes.

### Fish and feeding trial

Loaches (initial body weight: 0.44±0.01 g, mean±SE) here were obtained from artificial reproduction by our laboratory and acclimated to laboratory conditions for 2 weeks by feeding a commercial feed (35% crude protein and 10% crude lipid, Haida Co. Ltd.). The growth trial lasted for 12 weeks and was conducted indoors in a system with flow-through fresh water at 1 L/min. Three tanks (50 L water/tank) were randomly assigned to each dietary treatment with 20 loaches per tank. All loaches were hand-fed to apparent satiation with the designated diets thrice daily (8:00, 14:00 and 20:00 h). A diurnal cycle of 12/12 h light/dark was maintained with fluorescent lights. Water temperature ranged from 20°C to 24°C, pH fluctuated between 7.0 and 7.5, and DO was maintained approximately at 6.5 mg/L during the feeding trial.

### Sample collection and preparation

After 12 weeks feeding trial, loaches from the three different groups (i.e. FO group, MO group and HO group) were fasted for 24 h. All loaches in each tank were anesthetized with 100 mg/L MS-222 and weighed to determine their final body weights. The livers of three loaches from each tank were dissected out and fixed in phosphate buffered saline (PBS) containing 4% paraformaldehyde (PFA) at 4°C for oil red O staining. Five loaches from each tank were randomly collected. Their livers were dissected out and rapidly frozen in liquid nitrogen for 24 hours immediately, and then stored at -80°C for RNA extractions.

### Oil red O staining

After fixed in PBS containing 4% PFA at 4°C for one week, frozen liver tissue was sectioned (5 μm) and committed to lipid staining with oil red O following the method of Gen *et al* [29].

### RNA extraction, cDNA library and illumina sequencing

Total RNA was extracted from the livers of five loaches from each group using Trizol (Invitrogen, CA, USA) according to the manufacturer’s instructions. The concentration of total RNA was determined by NanoDrop 2000 (Thermo Scientific, Waltham, MA), and the RNA integrity value was checked by the RNA 6000 Pico LabChip on an Agilent 2100 Bioanalyzer (Agilent, Santa Clara, CA). To obtain purified mRNA, total RNA was incubated with 10 U DNase I (Ambion, Grand Island, NY) at 37°C for 1 h, followed by a purification step using a MicroPoly (A) Purist Kit (Ambion, Grand Island, NY) according to the manufacturer’s instructions. The total mRNAs extracted from the livers of each group were pooled together as one group-specific sample. Poly (A) mRNA was separated using oligo-dT beads (Qiagen, Dusseldorf, Germany). The fragmentation buffer was added to split all mRNA into short fragments. Random hexamer-primed reverse transcription was used for the first-strand cDNA synthesis. RNase H and DNA polymerase I for following generation of the second-strand cDNA were used. The QIAquick PCR extraction kit was performed to purify the cDNA fragments. These purified cDNA fragments were washed by EB buffer for end reparation poly (A) addition and then ligated to sequencing adapters. After that, agarose gel electrophoresis was used to separate the short fragments. The fragments with a size suitable for sequencing criteria were isolated from the gels and enriched by PCR amplification to construct the final cDNA library. Thereafter, the cDNA library was sequenced on the Illumina sequencing platform (Illumina HiSeq 2500) using the single-end paired-end technology in a single run, by Biomarker Technologies CO. LTD, Beijing, China. The Illumina GA processing pipeline was used to analyze the image and for base calling.

### *De Novo* assembly and functional annotation

High quality sequences were indispensable for *de novo* assembly analysis. Raw sequencing reads were clipped by discarding adapter sequences and ambiguous nucleotides before assembly. Then all clean reads of the libraries of the three different groups assembled into transcripts by Trinity software. Trinity is a modular method which combines three components: Inchworm, Chrysalis and Butterfly. Firstly, Inchworm assembles reads by a greedy k-mer based approach for linear contigs collection. Contigs longer than 200 bases were used for subsequent analysis. Chrysalis clusters the related contigs, and then a de Bruijn graph is built for each cluster. Finally, Butterfly analyzes the paths based on reads and read pairings from the corresponding de Bruijn graph and outputs full-length transcripts for alternatively spliced isoforms. After assembly, the TGICL clustering software (J. Craig Venter Institute, Rockville, MD, USA) was used to cluster and remove redundant transcripts, and then the remaining sequences were defined as unigenes. Blastx with an E-value <10^−5^ between the unigenes and the databases non-redundant proteins (Nr), Swiss-Prot, Kyoto Encyclopedia of Genes and Genomes (KEGG), Gene ontology (GO) and Clusters of Orthologous Groups (COG) was conducted. GO annotation of these unigenes was produced using Blast2GO based on the results of the NCBI Nr database annotation. Blastn was used for aligning these unigenes to the Nr database, searching proteins with the highest sequence similarity to the given unigenes, accompanied by their protein functional annotations.

### Analysis of Differentially Expressed Genes (DEGs)

The mapped reads were normalized according to fragment per kilobase of exon model per million mapped reads (FPKM) [30] for each unigene between the three pooled samples (i.e. FO, MO and HO group), which facilitated the comparison of unigene expression between samples. Differentially expressed genes (DEGs) between the two groups (MO_vs_FO, HO_vs_FO and MO_vs_HO) were identified by the DEGseq package applying the MA-plot-based method with Random Sampling model (MARS) method. We used false discovery rate (FDR) to determine the threshold of p value for this analysis. FDR (false discovery rate) <0.01 and the absolute value of log_2_ Ratio >1 was considered to have significant expression abundance. Each DEG between every two groups should be at least two-fold. COG annotation of the DEGs was performed using Blastall software. Go enrichment analysis (p-value ≤ 0.05) of the DEGs was performed using GOseq with the Wallenius non-central hyper-geometric distribution model to adjust gene length bias in DEGs. KEGG pathway enrichment analysis of the DEGs was done using KOBAS with the hyper-geometric distribution model. The enrichment p-values were adjusted using the Benjamin and Hochberg method.

### Quantitative real time PCR for RNA-seq results validation

Fourteen genes were randomly selected for validation of the RNA-seq results by quantitative real time PCR (qPCR) using the SYBR Premix Ex Taq kit (Takara, Japan) according to the manufacturer’s instructions. RNA samples were treated by RQ1 RNase-Free DNase (Takara Co. Ltd, Japan) prior to qPCR to avoid genomic DNA amplification. cDNA was generated from 500ng DNase-treated RNA using ExScript RT-PCR kit (Takara Co. Ltd, Japan). Primers (S2 Table) for qPCR analysis were designed using the Primer 5 Software. qPCR was performed in a Mini Option real-time detector (Bio-Rad, USA). The qPCR reaction solution consisted of 5μl SYBRpremix Ex Taq (2×), 0.4μl PCR forward primer (20μM), 0.4μl PCR reverse primer (10μM), 1.0μl RT reaction (cDNA solution), and 3.2μl dH_2_O. The reaction conditions were as follows: 95°C for 30s followed by 40 cycles consisting of 95°C for 5 s and 57°C for 30s. The florescent flux was then recorded, and the reaction continued at 72°C for 6s and 95°C for 5s. All amplicons were initially separated by agarose gel electrophoresis to ensure that they were of correct sizes. The gene expression levels were normalized towards the mean of the two reference genes (*β-Actin* and *glyceraldehyde-3-phosphate dehydrogenase*). Normalized gene expressions of the FO group were set to 1, and the expressions of each target gene of MO and HO group were expressed relative to the FO group. Optimized comparative Ct (2^-ΔΔCt^) value method was used here to estimate gene expression levels. Data were expressed as means of three parallel experiments performed.

## Results

### Growth performance and oil red o staining

**Fig 1:**
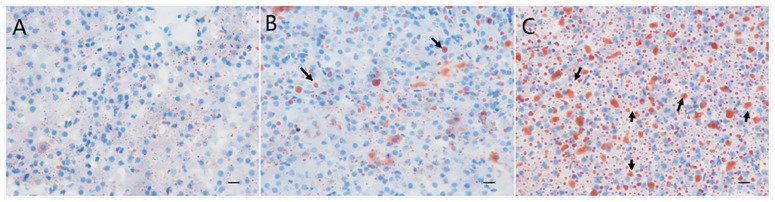
Lipid distributions in livers of loaches from FO group (A), MO group (B) and HO group (C).

There were no significantly differences in body weight gain among FO, MO, and HO group. Few lipid droplets appeared in livers of loaches from FO (Fig 1A) and MO group (Fig 1B), while a large number of lipid droplets clusters were observed in HO group (Fig 1C). The number of lipid droplets in loach liver became bigger and bigger along with increasing of the oxidized degree of the dietary fish oil.

### Transcriptome sequencing and statistics of unigenes

To obtain an overview of the hepatic transcriptome of loach fed with different oxidation degrees of dietary fish oils, cDNA samples of the livers for FO, MO and HO group were mixed respectively and sequenced on an Illumina machine. A total of 15.81Gb clean data was obtained, while 26,287,518, 27,604,024, and 25,145,839 clean reads were from FO, MO and HO group, respectively. These raw data were assembled into 9,938,514 contigs in total. Length distributions of these contigs and unigenes are shown in S1 Fig. The mix assembly of the three transcriptomes produced a substantial number of large contigs: 14,884 contigs were larger than 1,000 bp and 26,452 contigs larger than 500 bp. Among the 60,663 unigenes in total, 14,654 were larger than 1,000 bp.

**Fig 2:**
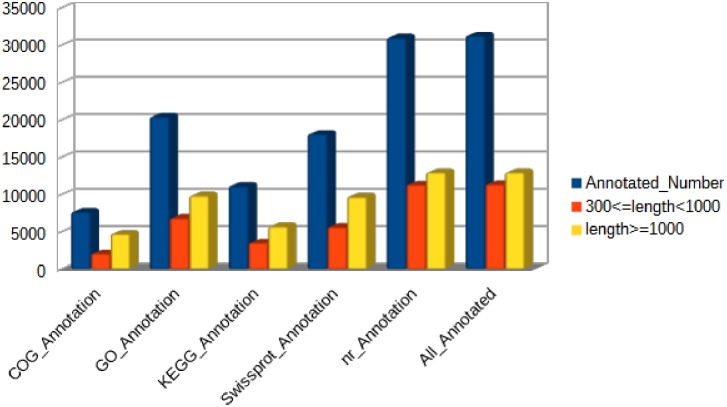
Unigenes annotated by five public databases (COG, GO, KEGG, Swissprot and Nr).

Approximately 51.5% of the unigenes (31,269) were annotated by Blastx and Blastn against five public databases (Nr, Swiss-Prot, KEGG, COG and GO). Among these unigenes, 31,026, 18,106, 11,135, 7,667 and 20,441 were identified in the Nr, Swiss-Prot, KEGG, COG and GO databases, respectively (Fig 2).

**Fig 3:**
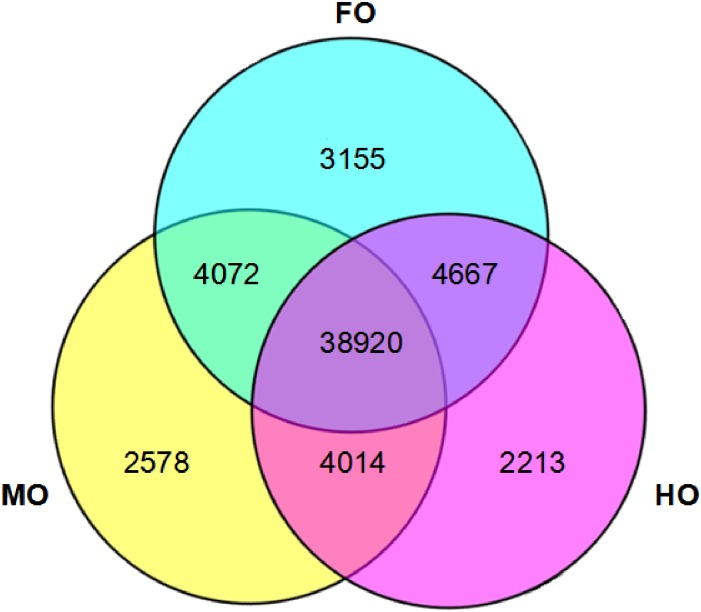
A venn diagram showing the number of unigenes in FO, MO and HO group.

As shown in the Fig 3, there were 50,814, 49,584 and 49,814 unigenes in FO, MO and HO respectively. Meanwhile, 38,920 unigenes were obtained in the three groups commonly; 4072, 4667 and 4014 were shared by every two groups, namely FO and MO, FO and HO, MO and HO, respectively; 3155, 2587 and 2213 unigenes were specifically expressed in FO, MO and HO group, respectively.

### Functional annotation and classification of unigenes

All unigenes from livers of the loaches were used for function enrichment and classifications analysis. COG is a classification system based on orthologous genes. Orthologous genes have the same function and common ancestor. Together, 7,667 (24.51%) unigenes were annotated in COG and grouped into 25 COG classifications (S2 Fig). The largest cluster was the general function prediction only, which meant that most of the genes’ functions were predicted by informatics methods and has not been confirmed by experimentation, and then followed by replication, recombination and repair, signal transduction mechanisms and transcription.

Through alignment of GO database, 20,441 (65.37%) unigenes were annotated to 51 terms of GO classification (S3 Fig). For the cellular component, cell part (11,448 unigenes), cell (11,250 unigenes), organelle (8,413 unigenes) and membrane (6,499 unigenes) represented the majorities of this category. Binding (11,630 unigenes) and catalytic activity (7,508 unigenes) represented a high percentage of the molecular function category. Moreover, cellular process (13,057 unigenes), metabolic process (9,481 unigenes) and biological regulation (8,720 unigenes) represented the majorities of the biology process.

### Identification of differentially expressed genes

Read count data obtained from the transcriptome was used to analyze the differences of gene expression. There were 2,343 DEGs identified in the comparison of MO_vs_FO, with 855 down- and 1,488 up-regulated genes in MO group; 2,813 DEGs in HO_vs_FO, with 1,256 down- and 1,552 up-regulated genes in HO group; 2,075 DEGs in MO_vs_HO, with 1,074 up- and 1,001 down-regulated genes in MO group. The numbers of DEGs between the three pairwise comparisons are shown in Table 1. We found that the comparison of HO_vs_FO had the most specific differentially expressed genes. Interestingly, MO_vs_FO and HO_vs_FO had 1285 common differentially expressed genes.

**Table 1:** The summary of the numbers of DEGs.

DEG set	All DEGs	Down-regulated	Up-regulated
MO_vs_FO	2,343	855	1,488
HO_vs_FO	2,813	1,261	1,552
MO_vs_HO	2,075	1,001,	1,074

FO, fresh fish oil diet; MO, medium oxidized fish oil diet; HO, high oxidized fish oil diet; DEG, differentially expressed gene.

### Enrichment of functional analysis of differentially expressed genes

Differentially expressed genes from the DEGseq analysis were further analyzed using COG, GO and KEGG enrichment in order to determine their potential functions and metabolic pathways.

**Fig 4:**
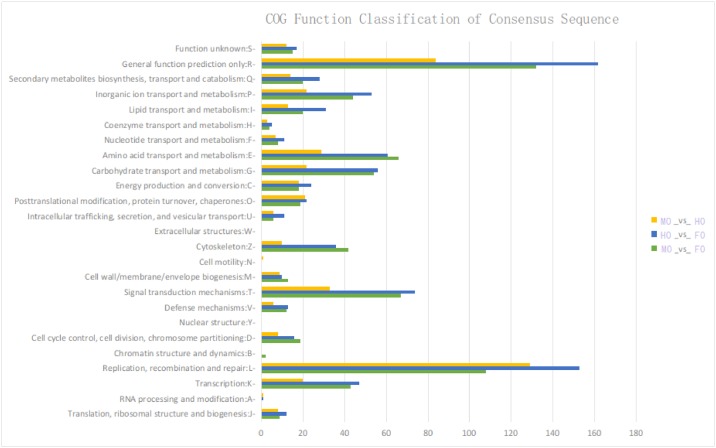
COG annotations of differentially expressed genes in comparisons of MO_vs_FO, HO_vs_FO and MO_vs_HO.

COG enrichment analysis of the DEGs is shown in Fig 4. Replication, recombination and repair (L) annotated the most DEGs in the comparison of MO_vs_HO and general function predict only (R) annotated the most both in MO_vs_FO and HO_vs_FO, while nuclear structure (Y), extracellular structures (W) and cell motility (N) annotated the least DEGs in all the three comparisons.

**Fig 5:**
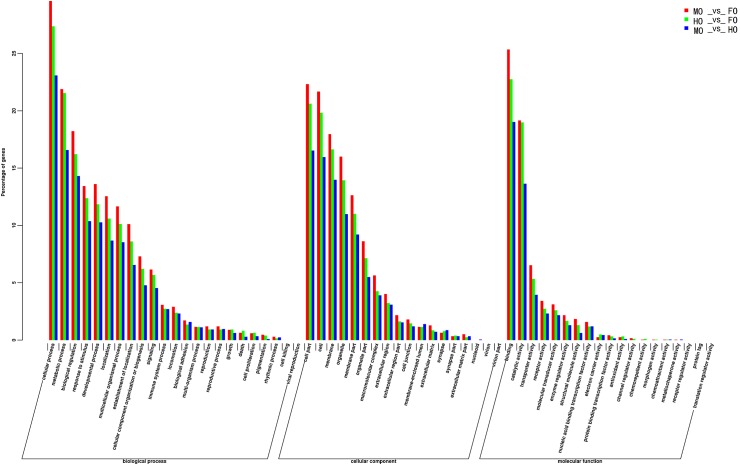
GO classifications of the differentially expressed genes in comparisons of MO_vs_FO, HO_vs_FO and MO_vs_HO.

Fig 5 shows GO classifications of the DEGs in comparisons of MO_vs_FO, HO_vs_FO and MO_vs_HO. Binding, cell part and cellular process were the most abundant GO function items (>15%) in the three comparisons. In the biological process, cellular process, metabolic process and biological regulation had the most abundant GO function items in the pairwise comparisons while cell, cell part and membrane had the most in the cellular component. In the molecular function, followed by binding and catalytic activity which had the most abundant GO function items, transporter activity, receptor activity and molecular transducer activity had more GO items than the others.

**Fig 6:**
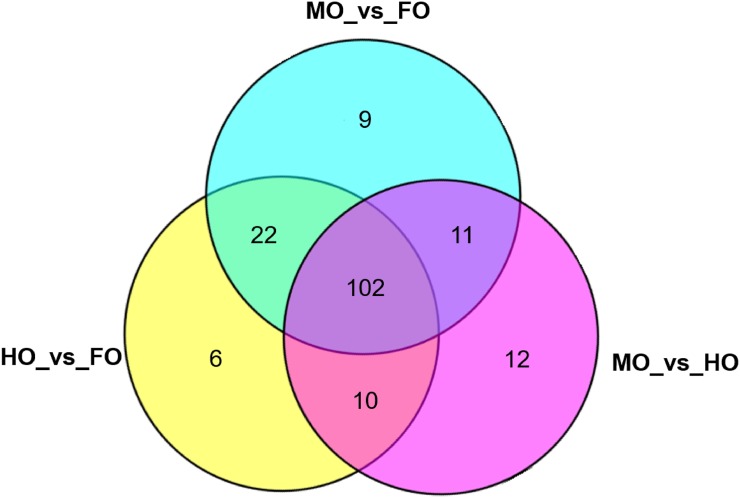
A venn diagram showing the numbers of KEGG pathways of the differentially expressed genes in comparisons of MO_vs_FO, HO_vs_FO and MO_vs_HO.

Pathway analysis was performed using mapped loach DEGs to mammalian orthologues to get more insight into the effects of dietary oxidized fish oil on metabolisms in the loach. In total, the DEGs obtained in MO_vs_FO were mapped into 144 pathways, and 140 pathways in HO_vs_FO. In addition, the DEGs in the comparison of MO_vs_HO were mapped into 136 pathways. As shown in Fig 6, 102 pathways were commonly performed in the comparisons of every two groups. Usually, the pathways were performed using complete DEGs lists or two separated lists of DEGs representing up- and down-regulation of genes. But better enrichment of DEGs in their supporting pathways was observed when using complete DEGs rather than separated lists. Our KEGG pathway enrichment analysis of the DEGs showed that many lipid metabolism-related pathways were significantly enriched in DEGs after feeding oxidized fish oil (S3 Table).

**Fig 7:**
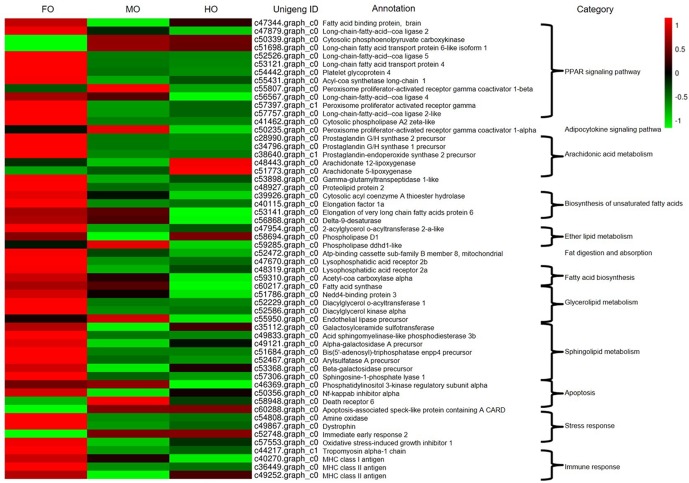
Heat map of expression levels of DEGs related with lipid metabolism of liver in the loach.

Many genes related to PPARs signaling pathway, arachidonic acid metabolism, sphingolipid metabolism, fat digestion and absorption, glycerolipid metabolism, ether lipid metabolism, biosynthesis of unsaturated fatty acids and fatty acid biosynthesis were detected in the comparisons of every two groups, meanwhile some genes involved in the stress response, apoptosis and immune response were also found (Fig 7). Compared to the FO group, lots of genes in MO and HO groups associated with PPARs signaling pathway (*fatty acid transport protein* (*fatp*), *fatty acid binding protein* (*fabp*), *apolipoprotein* (*apo*), *acyl-CoA synthetase* (*acs*), *peroxisome proliferator-activated receptor-γ* (*ppar-γ*), etc.), arachidonic acid metabolism (*prostaglandin G/H synthase 2 precursor*, *prostaglandin G/H synthase 1 precursor*, *prostaglandin-endoperoxide synthase 2 precursor*, etc.), and sphingolipid metabolism (*galactosylceramide sulfotransferase*, *alpha-galactosidase A precursor*, *bis* (*5'-adenosyl*)*-triphosphatase enpp4 precursor*, *sphingosine-1-phosphate lyase 1*, etc.), as well as stress response (*amine oxidase* (*amo*)), immune response (*tropomyosin alpha 1* (*αTM1*)) and apoptosis (*NF-kappaB inhibitor alpha* (*NF-kBIα*)) were down-regulated. 8 out of 8 DEGs encoding arachidonic acid metabolism pathway were down-regulated in MO_vs_FO and 7 out of 11 DEGs down-regulated in HO_vs_FO, while *arachidonate 5-lipoxygenase* (*alox5*) and *arachidonate 12-lipoxygenase* (*alox12*) were up-regulated in the comparison of HO_vs_FO.

### Validation of RNA-Seq results by quantitative real time PCR

**Fig 8:**
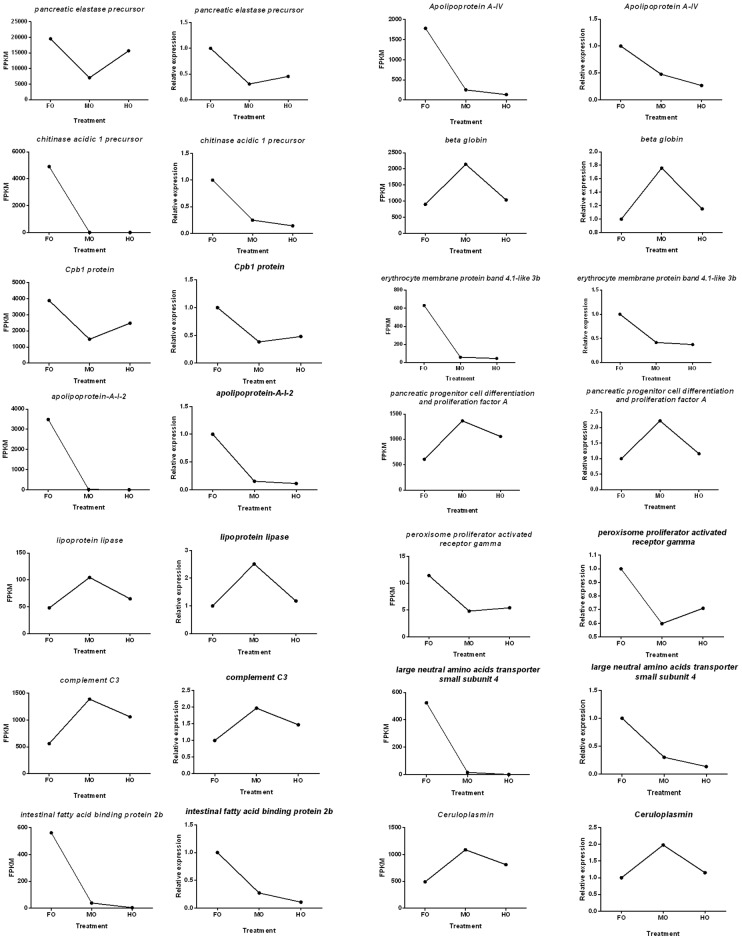
Candidate unigene expression levels revealed by qRT-PCR (right side) and RNA-seq (left side).

qPCR was performed on 14 randomly selected genes (including *pancreatic elastase precursor*, *chitinase acidic 1 precursor*, *Cpb1 protein*, *partial*, *apolipoprotein-A-I-2*, *Apolipoprotein A-IV*, *beta globin*, *erythrocyte membrane protein band 4*.*1-like 3b*, *pancreatic progenitor cell differentiation and proliferation factor A*, *complement C3*, *intestinal fatty acid binding protein 2b*, *large neutral amino acids transporter small subunit 4*, *Ceruloplasmin*, *lipoprotein lipase and peroxisome proliferator activated receptor gamma*) for validating the RNA-Seq results. The expression patterns revealed by qPCR analysis were similar to those revealed by RNA-Seq for the same genes (Fig 8). Above all, qPCR analysis confirmed the tendency detected by the mRNA sequencing analysis.

## Discussion

Fish oil has been the major source of lipid for the manufacture of fish aquafeeds. However, easy to be oxidized was the most intractable problem of fish oil [31]. A number of studies about dietary oxidized fish oil have been carried out in many fish species [32–34]. But no studies were about transcriptome profiles of fish which fed with dietary oxidized lipid let alone oxidized fish oil. In the present study, we are the first one seeking to identify genes response to dietary oxidized fish oil in loach *M*. *anguillicaudatus* through transcriptome analysis, which might become a molecular model of dietary oxidized lipid researches in fish. In total, 60,663 unigenes with a mean size of 848.74 bp were obtained from three groups with different oxidized levels of dietary fish oil (namely FO, MO, HO group). 31,269 unigenes were annotated to public protein databases altogether. Some DEGs involved in lipid metabolism were found here responding to dietary oxidized fish oil, which would benefit researches of lipid metabolism in the loach.

Fish oil, which contains a large amount of long chain polyunsaturated fatty acids, is susceptible to lipid peroxidation, where oxygen attacks the double bond in the fatty acids to form lipid. Meanwhile, long chain fatty acids function in physiology as energetic substrates, membrane components and signaling molecules by targeting nuclear receptors [35]. As members of the nuclear receptor superfamily of ligand-dependent transcription factors, peroxisome proliferator receptors (PPARs) control transcriptional rate of a large panel of genes implicated in organogenesis, cell proliferation, cell differentiation, inflammation and metabolism of lipids or carbohydrates once activated by their respective ligands (namely fatty acids or fatty acid metabolites) [36]. Our KEGG pathway enrichment analysis of the DEGs showed that the PPAR signaling pathway was significantly enriched in DEGs after feeding oxidized fish oil. PPAR signaling pathway (ko03320) was the first highest representation of the DEGs in comparisons of MO_vs_FO and HO_vs_FO, and it was the second highest in MO_vs_HO. The down-regulated DEGs coexisted in MO_vs_FO and HO_vs_FO, included *fatp*, *fabp*, *apo*, *ppar-γ* and *acs*. *fatp* transport activity is specific for long chain fatty acids since uptake of fatty acids shorter than 10 carbon atoms is unaffected by *fatp* expression [37,38]. *fabp* is responsible for the intake alongside with the transport of polar lipids like fatty acids [39]. Apolipoproteins are more likely to be related to the transference of structural lipids than to lipids used for energy purposes and *apo* is of great use of trafficking of structural lipids for new cell membranes, or signaling among others [40]. *ppar-γ* plays a crucial role in adipogenesis [41]. *acs* is responsible for synthesis cytosolic acetyl-CoA which is key precursor for fatty acid building [42]. These suggested that *fatp*, *fabp*, *apo*, *ppar-γ* and *acs* in the loach were responsive to dietary oxidized fish oil, indicating the dietary oxidized fish oil can obviously influence the lipid metabolism of the loach.

Arachidonic acid (ARA, 20:4n-6) is a long chain polyunstatured fatty acid that along with DHA are indispensable for growth and development during the perinatal period in humans [43]. In higher vertebrates, ARA is the major long chain polyunststured fatty acids in the central nervous tissue [44]. ARA displays well-recognized functional roles, acting as cell signalling molecule, either in its own right or after its conversion into oxidized derivatives (eicosanoids) [45]. Our KEGG pathway enrichment analysis of the DEGs showed that the arachidonic acid metabolism pathway (ko00590) was also significantly enriched in DEGs after feeding oxidized fish oil. Arachidonic acid metabolism pathway was the second highest representation of the DEGs in comparison of HO_vs_FO. Most DEGs encoding arachidonic acid metabolism pathway were down-regulated in HO_vs_FO, while *alox5* and *alox12* were up-regulated. *alox5* catalyzes ARA to form 5-hydroperoxyeicosatetraenoic acid and subsequently metabolises to leukotrienes [46], which are very active even at low physiological concentrations and play critical roles in the regulation of several biological processes [47,48]. It indicated that *alox5* and *alox12* may be sensitive to dietary oxidized fish oil.

Sphingolipid metabolism (ko00600) was also significantly enriched in DEGs after feeding oxidized fish oil. Sphingolipids aggregate with other lipids to form structures that regulate the transport of molecules across the membrane or mediate the transmission of signals [49–51]. It suggested that the dietary oxidized fish oil may affect the lipid structures of loach. Other functions of the sphingolipids include apoptosis [52] and regulation of cellular growth [53,54]. Therefore, it indicated that the dietary oxidized fish oil might cause some harms in the level of cell.

This study showed *amo* which is related to stress response [55] down-regulated after feeding the dietary oxidized fish oil. *αTM1* which is responsible for the cell shape and cell adhesions, as well as immunofluorescence localization of myofibril assembly markers [56] down-regulated in MO and HO group. *NF-kBIα* which may relate to cell cycle, cell and tissue differentiation [57] down-regulated after intaking the dietary oxidized fish oil. These indicated that *amo*, *αTM1* and *NF-kBIα* in the loach, which were respectively associated with stress response, immune response and apoptosis, were responsive to dietary oxidized fish oil.

## Conclusions

In summary, we reported a hepatic transcriptome of loach *M*. *anguillicaudatus*, fed with three different diets, namely fresh fish oil diet, medium oxidized fish oil diet and high oxidized fish oil diet. It is a valuable resource for future studies of loach genomics and will also benefit researches in other closely related species with significantly aquaculture importance. More importantly, this study identified some important genes (such as *fatp*, *fabp*, *apo*, *ppar-γ*, *acs* and *alox5*) response to dietary oxidized fish oil, which would benefit researches of lipid metabolism in the loach.

## Supporting information

S1 Table Formulations (%), proximate compositions (%) and peroxide values (POV meq/kg) of the three different diets. (DOC)

S2 Table Primer sequences of the candidate genes for qPCR. (DOC)

S3 Table Top 10 KEGG pathways related to lipid metabolism of loach *Misgurnus anguillicaudatus*. (DOC)

S1 Fig The length distributions of assembled contigs (A) and unigenes (B) of loach *Misgurnus anguillicaudatus*. (DOC)

S2 Fig The COG classifications of all unigenes from liver of loach *Misgurnus anguillicaudatus*. (DOC)

S3 Fig The GO classifications of all unigenes from liver of loach *Misgurnus anguillicaudatus*. (DOC)
