# Supplementary material for: Hepatic transcriptome analysis and identification of differentially expressed genes response to dietary oxidized fish oil in loach Misgurnus anguillicaudatus
Source: PLoS One. 2017 Feb 17;12(2):e0172386. doi: 10.1371/journal.pone.0172386 (PMC5315305; doi:10.1371/journal.pone.0172386)
Supplement: S1 Table — (DOC) [file pone.0172386.s001.doc]

**S1 Table. Formulations (%), proximate compositions (%) and peroxide values (POV meq/kg) of the three different diets**

| **Ingredient** | **FO** | **MO** | **HO** |
| --- | --- | --- | --- |
| Defatted fish meal ^a^ | 40 | 40 | 40 |
| Soybean concentrate | 10 | 10 | 10 |
| Wheat meal | 10 | 10 | 10 |
| Casein | 10 | 10 | 10 |
| α-Starch | 8 | 8 | 8 |
| Dextrin | 8 | 8 | 8 |
| α-cellulose | 1.5 | 1.5 | 1.5 |
| Fish oil ^b^ | 10 | 10 | 10 |
| Vitamins ^c^ | 1 | 1 | 1 |
| Minerals ^d^ | 1 | 1 | 1 |
| Vitamin C | 0.5 | 0.5 | 0.5 |
| *Proximate composition* |  |  |  |
| Moisture | 6.9 | 6.9 | 11.7 |
| Crude lipid (dry mass) | 34.0 | 36.4 | 34.0 |
| Crude protein (dry mass) | 47.0 | 47.8 | 46.7 |
| Ash (dry mass) | 9.1 | 9.2 | 9.2 |
| POV | 4.8 | 46.3 | 96.0 |

FO: fresh fish oil diet; MO: medium oxidized fish oil diet; HO: high oxidized fish oil diet; POV: peroxide value.

^a^ Defatted by 100% Ether.

^b^ Riken Vitamin, Tokyo, Japan.

^c^ Vitamin mixture (mg/kg diet): β-carotene, 32.12 mg; vitamin C, 230 mg; Vitamin D_3_, 3.24 mg; Menadione NaHSO_3_·3H_2_O (K_3_), 15.28 mg; Thiamine-Nitrate (B_1_), 19.24 mg; Riboflavin (B_2_), 64.12 mg; Pyridoxine-HCl (B_6_), 15.28 mg; Cyanocobalamine (B_12_), 0.04 mg; d-Biotin, 1.92 mg; Inositol, 1283.04 mg; Niacine (Nicotic acid), 256.56 mg; Ca Panthothenate, 89.34 mg; Folic acid, 4.8 mg; Choline choloride, 2623.12 mg; ρ-Aminobenzoic acid, 127.76 mg.

^d^ Mineral mixture (mg/kg diet): MgSO_4_, 3380 mg; Na_2_HPO_4_, 2153.33 mg; K_2_HPO_4_, 5913.33 mg; Fe Citrate, 733.33 mg; Ca Lactate, 8060 mg; Al(OH)_3_, 6.67 mg; ZnSO_4_, 86.67 mg; CuSO_4_, 2.67 mg; MnSO_4_, 20 mg; Ca(IO_3_)_2_, 6.67 mg; CoSO_4_, 26.67 mg.

Values represent means of three replicates
