# Supplementary material for: Hepatic transcriptome analysis and identification of differentially expressed genes response to dietary oxidized fish oil in loach Misgurnus anguillicaudatus
Source: PLoS One. 2017 Feb 17;12(2):e0172386. doi: 10.1371/journal.pone.0172386 (PMC5315305; doi:10.1371/journal.pone.0172386)
Supplement: S2 Table — (DOC) [file pone.0172386.s002.doc]

**S2 Table Primer sequences of the candidate genes for qPCR**

| **Gene** | **Forward primer (5’-3’)** | **Reverse primer (5’-3’)** | **Length (bp)** |
| --- | --- | --- | --- |
| *Pancreatic elastase precursor* | CAGAGAACTTGGCGTGTTGT | GCAGGAGAGCGATGTCATATC | 145 |
| *Chitinase acidic 1 precursor* | TCCACTGTCCAAGTTGCTCTT | TCTGCCAAGGTTGCTTCTGT | 168 |
| *Cpb1 protein, partial* | AGTGACACCTACTGCGGAAG | TGGACTTGTTGCTGCGAATG | 82 |
| *Apolipoprotein-A-I-2* | GCTGCCTTGTAGTGCTCCA | TTACCTCTGCCGTCCAATCTC | 159 |
| *Apolipoprotein A-IV* | GGAGGTGATTGCTGTAATGTAA | TTCCAAGTACTGCCAGTATGTA | 132 |
| *Beta globin* | TGGCTACGCATACGGTGAT | ATCGTGTATCCTTGGACTCAGA | 250 |
| *Erythrocyte membrane protein band 4.1-like 3b* | CGCTGTTGTATCCGCTGTC | ATGTTGCTGGTGTGCTTGTAA | 270 |
| *Pancreatic progenitor cell differentiation and proliferation factor A* | CACACGGTTGACACTTAGAGG | GGAACTGTTGCTGGATGTAGAT | 116 |
| *Complement C3* | AGAAGCAACCTAGTCAGATGGA | GGCTGAGCACTGATAGAATCG | 133 |
| *Intestinal fatty acid binding protein 2b* | CTCAGCCACATCAGCGTATAG | TTCTCACTGCGGTCCACTT | 166 |
| *Large neutral amino acids transporter small subunit 4* | CATTCTCCTGATGGCAACTGT | CGGCATAACTCCACCTATTCAA | 288 |
| *Ceruloplasmin* | TCATCAGAGCAGAGGAAGAGG | GATAGGTGGTGTCAGGAAGGA | 231 |
| *Lipoprotein lipase* | GAGCCGACGAGCGAGTCTACAAC | CAGTGACCGTCCATCCGTGAATA | 215 |
| *Peroxisome proliferator activated receptor gamma* | CTGGCTTTCACTATGGCGTTCA | TGGCATTTGTTGCGACTCTTCT | 183 |
| *β-actin* | CCGGCCCATCCATCGTCCAC | CTGCTGCATGGCCAGGTATGGT | 138 |
| *Glyceraldehyde-3-phosphate dehydrogenase* | ACCAACTGCTTGGCTCCCC | GGAATGACTTTGCCCACG | 253 |
