# Supplementary material for: Hepatic transcriptome analysis and identification of differentially expressed genes response to dietary oxidized fish oil in loach Misgurnus anguillicaudatus
Source: PLoS One. 2017 Feb 17;12(2):e0172386. doi: 10.1371/journal.pone.0172386 (PMC5315305; doi:10.1371/journal.pone.0172386)
Supplement: S3 Table — (DOC) [file pone.0172386.s003.doc]

**S3 Table Top 10 KEGG pathways related to lipid metabolism of loach *Misgurnus anguillicaudatus***

|  | **Pathway** | **DEGs with pathway annotation (340)** | **All genes with pathway annotation (6261)** | | ***p*_value** | **Pathway ID** |
| --- | --- | --- | --- | --- | --- | --- |
| MO_vs_FO | | | | | | |
| 1 | PPAR signaling pathway | 13 (3.82%) | | 70 (1.12%) | 8.05E-05 | ko03320 |
| 2 | Arachidonic acid metabolism | 8 (2.35%) | | 40 (0.64%) | 1.16E-03 | ko00590 |
| 3 | Sphingolipid metabolism | 8 (2.35%) | | 53 (0.85%) | 7.23E-03 | ko00600 |
| 4 | Glycosphingolipid biosynthesis - ganglio series | 2 (0.59%) | | 10 (0.16%) | 9.92E-02 | ko00604 |
| 5 | Fat digestion and absorption | 1 (0.29%) | | 2 (0.03%) | 1.06E-01 | ko04975 |
| 6 | Adipocytokine signaling pathway | 8 (2.35%) | | 96 (1.53%) | 1.49E-01 | ko04920 |
| 7 | Glycosphingolipid biosynthesis - lacto and neolacto series | 3 (0.88%) | | 27 (0.43%) | 1.79E-01 | ko00601 |
| 8 | Glycerolipid metabolism | 6 (1.76%) | | 75 (1.2%) | 2.21E-01 | ko00561 |
| 9 | Ether lipid metabolism | 3 (0.88%) | | 41 (0.65%) | 3.86E-01 | ko00565 |
| 10 | alpha-Linolenic acid metabolism | 1 (0.29%) | | 12 (0.19%) | 4.89E-01 | ko00592 |
| HO_vs_FO | | | | | | |
| 1 | PPAR signaling pathway | 15 (3.83%) | | 70 (1.12%) | 2.04E-05 | ko03320 |
| 2 | Arachidonic acid metabolism | 11 (2.81%) | | 40 (0.64%) | 2.23E-05 | ko00590 |
| 3 | Sphingolipid metabolism | 8 (2.04%) | | 53 (0.85%) | 1.63E-02 | ko00600 |
| 4 | Biosynthesis of unsaturated fatty acids | 4 (1.02%) | | 19 (0.3%) | 2.77E-02 | ko01040 |
| 5 | Fatty acid metabolism | 7 (1.79%) | | 50 (0.8%) | 3.47E-02 | ko00071 |
| 6 | Fatty acid biosynthesis | 2 (0.51%) | | 7 (0.11%) | 6.66E-02 | ko00061 |
| 7 | Glycerolipid metabolism | 8 (2.04%) | | 75 (1.2%) | 9.55E-02 | ko00561 |
| 8 | Fat digestion and absorption | 1 (0.26%) | | 2 (0.03%) | 1.21E-01 | ko04975 |
| 9 | Linoleic acid metabolism | 2 (0.51%) | | 14 (0.22%) | 2.17E-01 | ko00591 |
| 10 | Glycosphingolipid biosynthesis - lacto and neolacto series | 3 (0.77%) | | 27 (0.43%) | 2.37E-01 | ko00601 |
| MO_vs_HO | | | | | | |
| 1 | Linoleic acid metabolism | 3 (1.33%) | | 14 (0.22%) | 1.26E-02 | ko00591 |
| 2 | PPAR signaling pathway | 7 (3.1%) | | 70 (1.12%) | 1.26E-02 | ko03320 |
| 3 | Arachidonic acid metabolism | 5 (2.21%) | | 40 (0.64%) | 1.38E-02 | ko00590 |
| 4 | Ether lipid metabolism | 4 (1.77%) | | 41 (0.65%) | 5.91E-02 | ko00565 |
| 5 | Sphingolipid metabolism | 4 (1.77%) | | 53 (0.85%) | 1.23E-01 | ko00600 |
| 6 | Biosynthesis of unsaturated fatty acids | 2 (0.88%) | | 19 (0.3%) | 1.49E-01 | ko01040 |
| 7 | Fatty acid biosynthesis | 1 (0.44%) | | 7 (0.11%) | 2.27E-01 | ko00061 |
| 8 | Fatty acid metabolism | 3 (1.33%) | | 50 (0.8%) | 2.69E-01 | ko00071 |
| 9 | alpha-Linolenic acid metabolism | 1 (0.44%) | | 12 (0.19%) | 3.57E-01 | ko00592 |
| 10 | Adipocytokine signaling pathway | 4 (1.77%) | | 96 (1.53%) | 4.58E-01 | ko04920 |

FO: fresh fish oil diet; MO: medium oxidized fish oil diet; HO: high oxidized fish oil diet
