# Supplementary material for: Hepatic transcriptome analysis and identification of differentially expressed genes response to dietary oxidized fish oil in loach Misgurnus anguillicaudatus
Source: PLoS One. 2017 Feb 17;12(2):e0172386. doi: 10.1371/journal.pone.0172386 (PMC5315305; doi:10.1371/journal.pone.0172386)
Supplement: S1 Fig — (DOC) [file pone.0172386.s004.doc]

A:


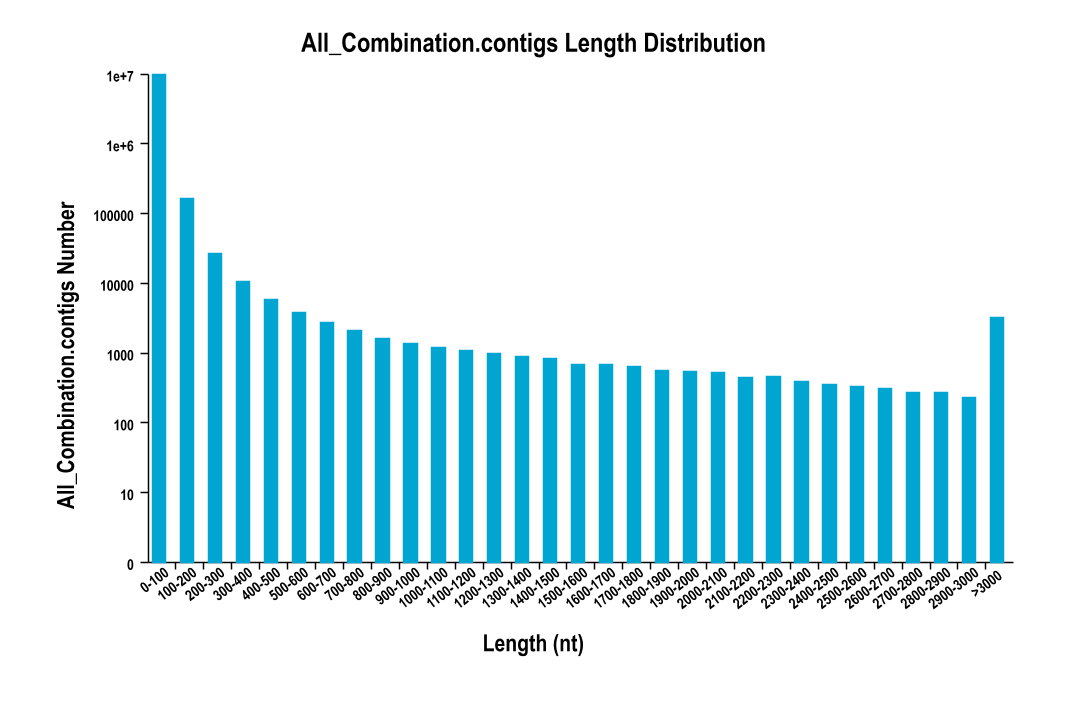


B:


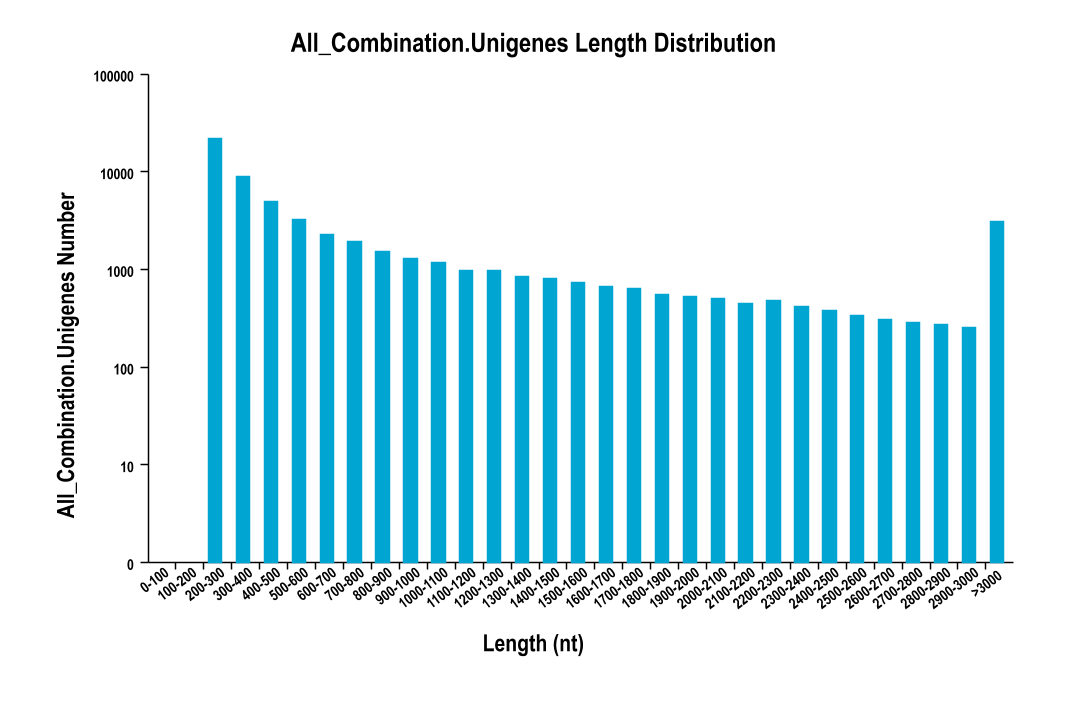


**S1 Fig. The length distributions of assembled contigs (A) and unigenes (B) of loach *Misgurnus anguillicaudatus*.**
