# Supplementary figures and images for: Hepatic transcriptome analysis and identification of differentially expressed genes response to dietary oxidized fish oil in loach Misgurnus anguillicaudatus
Source: PLoS One. 2017 Feb 17;12(2):e0172386. doi: 10.1371/journal.pone.0172386 (PMC5315305; doi:10.1371/journal.pone.0172386)

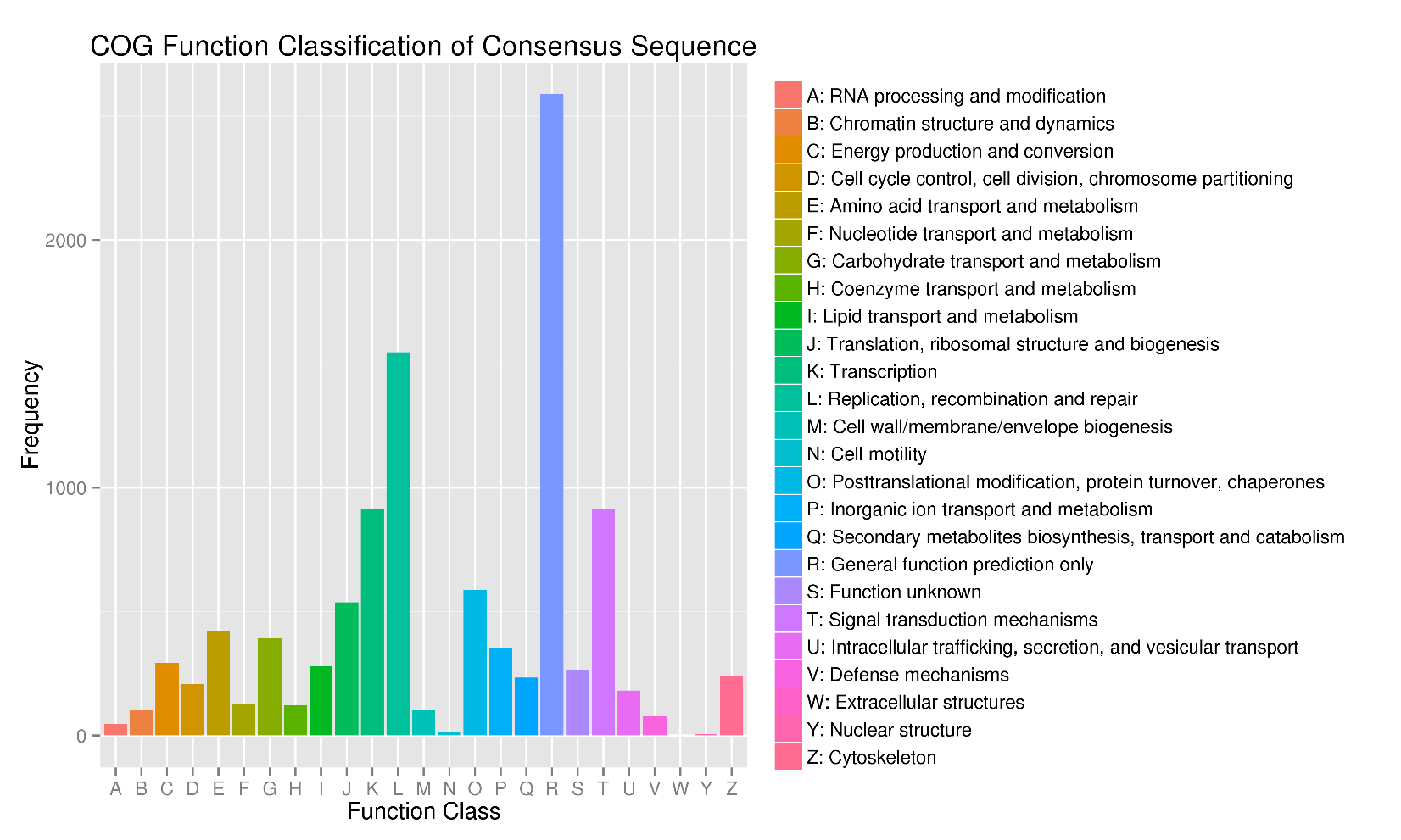


**S2 Fig.** **The COG classifications of all unigenes from liver of loach *Misgurnus anguillicaudatus***.

Supplement: S2 Fig — (DOC) [file pone.0172386.s005.doc]

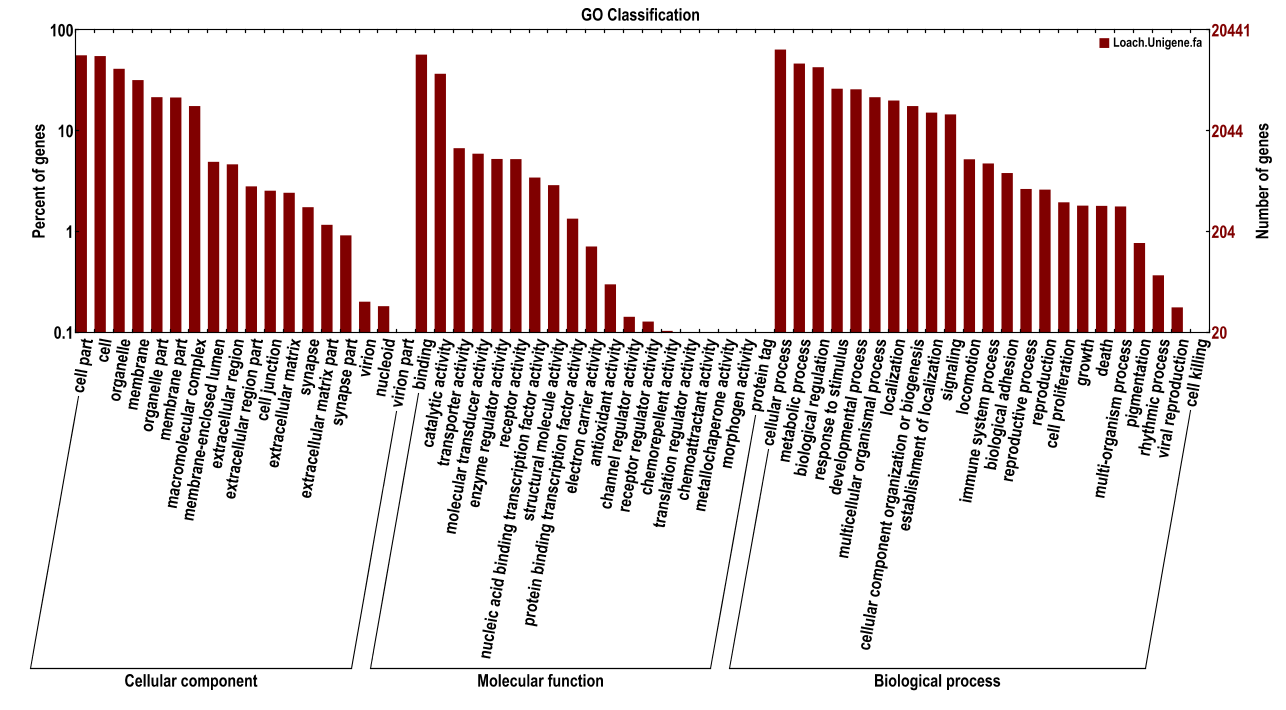


**S3 Fig. The GO classifications of all unigenes from liver of loach *Misgurnus anguillicaudatus*.**

Supplement: S3 Fig — (DOC) [file pone.0172386.s006.doc]
